# Methanogenic biodegradation of C_9_ to C_12_*n*-alkanes initiated by *Smithella* via fumarate addition mechanism

**DOI:** 10.1186/s13568-020-0956-5

**Published:** 2020-02-01

**Authors:** Jia-Heng Ji, Lei Zhou, Serge Maurice Mbadinga, Muhammad Irfan, Yi-Fan Liu, Pan Pan, Zhen-Zhen Qi, Jing Chen, Jin-Feng Liu, Shi-Zhong Yang, Ji-Dong Gu, Bo-Zhong Mu

**Affiliations:** 1 State Key Laboratory of Bioreactor Engineering and School of Chemistry and Molecular Engineering, East China University of Science and Technology, 130 Meilong Road, Shanghai, 200237 People’s Republic of China; 2 Department of Chemical, Polymer and Composite Materials Engineering, University of Engineering and Technology, KSK Campus, Lahore, 54890 Pakistan; 3 School of Biological Sciences, The University of Hong Kong, Pokfulam Road, Hong Kong, Special Administrative Region People’s Republic of China; 4 Engineering Research Center of Microbial Enhanced Oil Recovery, East China University of Science and Technology, 130 Meilong Road, Shanghai, 200237 People’s Republic of China

**Keywords:** Alkanes, Alkylsuccinates, Fumarate addition, Methanogenesis, Oil reservoirs

## Abstract

In the present study, a methanogenic alkane-degrading (a mixture of C_9_ to C_12_*n*-alkanes) culture enriched from production water of a low-temperature oil reservoir was established and assessed. Significant methane production was detected in the alkane-amended enrichment cultures compared with alkane-free controls over an incubation period of 1 year. At the end of the incubation, fumarate addition metabolites (C_9_ to C_12_ alkylsuccinates) and *assA* genes (encoding the alpha subunit of alkylsuccinate synthase) were detected only in the alkane-amended enrichment cultures. Microbial community analysis showed that putative syntrophic *n*-alkane degraders (*Smithella*) capable of initiating *n*-alkanes by fumarate addition mechanism were enriched in the alkane-amended enrichment cultures. In addition, both hydrogenotrophic (*Methanocalculus*) and acetoclastic (*Methanothrix*) methanogens were also observed. Our results provide further evidence that alkanes can be activated by addition to fumarate under methanogenic conditions.

## Introduction

Methanogenic biodegradation of crude oil is a prevalent process occurring in subsurface petroleum reservoirs and has adverse effect on oil quality (Head et al. 2003, 2006; Jones et al. 2008). However, it has been postulated that methanogenic crude oil degradation can be applied for energy recovery in depleted petroleum reservoirs by bioconversion of residual oil to methane (Gieg et al. 2008). In addition to energy recovery, methanogenic degradation of crude oil is also a major process for bioremediation in the oil-contaminated environments after the depletion of electron acceptors (Amos et al. 2005; Feisthauer et al. 2010, 2012).

*n*-Alkanes are the major constituents of crude oil and also the significant contaminants in oil-polluted environments. Methanogenic biodegradation of *n*-alkanes requires the initial activation of these substrates before the further degradation (Zengler et al. 1999). Alkane activation by homolytic cleavage of the C–H bond, followed by addition of the resulting radical to the double bond of fumarate with the formation of alkylsuccinates is the most ubiquitous anaerobic *n*-alkane activation mechanism (Callaghan 2013), which has been demonstrated under sulfate- (Kniemeyer et al. 2007; Kropp et al. 2000) and nitrate-reducing conditions (Rabus et al. 2001). Only a few studies proved fumarate addition occurred under methanogenic conditions with limited detection of initial metabolites alkylsuccinates. Toth and Gieg detected C_1_ to C_9_ alkylsuccinates and *assA* genes over the incubation time of the methanogenic crude oil-degrading enrichment cultures (Toth and Gieg 2017). Qin et al. identified C_15_ and C_16_ alkylsuccinates in methanogenic pentadecane- and hexadecane-degrading enrichment cultures, respectively (Qin et al. 2017). In our recent work, a series of C_16_ to C_20_ alkylsuccinates and *assA* genes were detected in the methanogenic enrichment cultures amended with C_16_ to C_20_*n*-alkanes (Ji et al. 2019).

Although methanogenic biodegradation of crude oil (Aitken et al. 2013; Gieg et al. 2010; Gray et al. 2011; Jones et al. 2008; Toth and Gieg 2017) and longer *n*-alkanes (≥ C_14_) (Liang et al. 2016; Siddique et al. 2011; Wawrik et al. 2016; Zengler et al. 1999; Zhou et al. 2012) has been intensively investigated, methanogenic biodegradation of low molecular weight *n*-alkanes has not been extensively studied (e.g. activation mechanisms and syntrophic degraders). Members of genus *Smithella* (in the family *Syntrophaceae*), implicated in syntrophic alkane degradation, were frequently identified in methanogenic crude oil-degrading enrichment cultures (Gray et al. 2011; Jones et al. 2008; Toth and Gieg 2017). Sherry et al. observed that *Smithella* was significantly enriched in both the weathered and non-weathered oil-amended (containing C_5_ to C_10_*n*-alkanes) methanogenic enrichment cultures, indicating that *Smithella* can utilize low molecular weight *n*-alkanes (Sherry et al. 2014). Novel members of the family *Peptococcaceae* were identified to be the primary degraders in several methanogenic short alkane-degrading (C_5_ to C_10_; *n*-, *iso*- and *cyclo*-alkanes) enrichment cultures derived from oil sands tailings ponds (Abu Laban et al. 2015; Mohamad Shahimin et al. 2016; Mohamad Shahimin and Siddique 2017; Siddique et al. 2015; Tan et al. 2014; Tan et al. 2013). By investigating methanogenic biodegradation of C_7_ to C_8_*iso*-alkanes, Abu Laban et al. proposed a novel family *Peptococcaceae* activated these substrates by addition to fumarate with the detection of high abundance of *Peptococcaceae*, *Peptococcaceae*-related *assA* gene and fumarate addition metabolites of C_7_ to C_8_*iso*-alkans (Abu Laban et al. 2015). Although a positive expression of *assA* gene and fumarate addition metabolites of 2-methylpentane and methylcyclopentane were detected in the methanogenic short alkane-degrading (C_6_ to C_10_; *n*-, *iso*- and *cyclo*-alkanes) cultures, Tan et al. still failed to detect initial activation metabolites of *n*-alkanes (Tan et al. 2015).

Here, we established a methanogenic enrichment culture growing on C_9_ to C_12_*n*-alkanes inoculated with production water from a low-temperature petroleum reservoir. Methane production was periodically monitored during the incubation. Microbial community compositions, functional genes (*assA* and *mcrA*) and metabolite profiles were analyzed at the end of the incubation period.

## Materials and methods

### Enrichment cultures

Production water from Xinjiang Kelamayi oil field block 6 (about 21 °C) was collected and stored in a serum bottle with headspace filled with N_2_ (99.99% purity). The production water was stored for over 1 year to consume the residual organics. Sterilized basal medium with no electron acceptor (Wang et al. 2011) was dispensed in 120 mL-serum bottles as 48 mL per each. 2 mL of the production water was transferred to each bottle by syringe. Each alkane-amended enrichment culture contained 0.225 mmol of each *n*-alkane, including *n*-nonane (C_9_; ≥ 99%), *n*-decane (C_10_; ≥ 99%), *n*-undecane (C_11_; ≥ 99%) and *n*-dodecane (C_12_; ≥ 99%). Alkane-free control cultures received no *n*-alkane. The serum bottles were sealed with butyl rubber stoppers. All the cultures were set up in two replications and stationarily incubated at room temperature (around 21 °C) in the dark.

### Methane measurements

Methane production was measured using a gas chromatography (GC model 9890B, Shanghai, China) equipped with a flame ionization detection (FID). 200 μL headspace gas taken by a gastight syringe were injected into GC for analysis. Program setting of the GC analysis was: the initial column temperature was set at 50 °C for 2 min, then increased to 130 °C at a rate of 15 °C/min, the temperature of 130 °C sustained for 1 min; the second increase was conducted at a rate of 30 °C/min to 180 °C for 30 min. The temperature of injector and FID was 200 °C. External standard curve of the methane was used for methane concentration calculation (Ma et al. 2018).

### Metabolites measurements

To detect acid metabolites in the cultures, about 40 mL of culture aliquots was collected. These culture aliquots were refluxed at 100 °C for 8 h with 50 mL 1 M KOH in a 50% methanol, 50% water mixture for saponification. This was followed by acidification to pH < 2 with HCl. The organic fraction was then extracted with ethyl acetate and derivatized to ethyl esters with 10 mL of ethanol, 10 mL of cyclohexane and 0.2 g of NaHSO_4_ (refluxed at 80 °C for 8 h). After rotary evaporation, 10 mL deionized water was added. Metabolites were extracted with 10 mL ethyl acetate for three times and concentrated to about 200 μL. 1 μL sample was injected into GC–MS in a splitless mode for analysis. An Agilent 7890A GC coupled to a MSD 5975C mass detector was used. The injector temperature was 280 °C. The program of GC–MS was followed: the initial temperature was held at 60 °C for 2 min, then increased at a rate of 10 °C/min to 280 °C for 20 min. The MS detector was run in the scan mode from 30 to 1000 mass units.

Diethyl (1-methyloctyl)succinate was synthesized according to Bian et al. (2014). The identification of (1-methyloctyl)succinate in the enrichment cultures was compared with the synthesized authentic standard (Additional file 1: Figure S1). (1-methylnonyl)succinate, (1-methyldecyl)succinate and (1-methylundecyl)succinate were identified by their characteristic fragment ions (128, 174, [M–45]^+^ and [M–87]^+^) (Bian et al. 2014) and relative retention times. Diethyl succinate, diethyl glutarate, diethyl adipate, diethyl suberate and diethyl azelate were synthesized by ethyl esterification of succinic acid, glutaric acid, adipic acid, suberic acid and azelaic acid, respectively. The reaction mixture contained 2 mg of dicarboxylic acid, 10 mL of ethanol, 10 mL of cyclohexane and 0.2 g of NaHSO_4_. The reaction mixture was refluxed at 80 °C for 8 h. The ethanol and cyclohexane were removed by rotary evaporation, and the residue was treated with 10 mL water. Diethyl products were extracted with extracted with 10 mL ethyl acetate for three times and analyzed by GC–MS in a same program of culture metabolites analysis. α,ω-Dicarboxylic acids in the enrichment cultures were identified by comparison with these authentic standards (Additional file 1: Figure S2). Fatty acids were identified by matching library spectra NIST (https://webbook.nist.gov/chemistry/).

### Microbial community analysis

10 mL of culture aliquot were collected for genomic DNA extraction using the AxyPrep™ Bacterial Genomic DNA Maxiprep Kit (Axygen Biosciences, USA). Archaeal and bacterial 16S rRNA genes were amplified using 344F (5′-ACGGGGYGCAGCAGGCGCGA-3′)/915R (5′-GTGCTCCCCCGCCAATTCCT-3′) (Casamayor et al. 2002) and 515F (5′-GTGCCAGCMGCCGCGG-3′)/907R (5′-CCGTCAATTCMTTTRAGTTT-3′) (Xiong et al. 2012), respectively. 16S rRNA gene polymerase chain reaction (PCR) and Illumina sequencing were performed as previously described (Ma et al. 2018). Operational taxonomic units (OTUs) were classified using Usearch (Edgar 2013) against the SILVA SSU database 128 (Quast et al. 2013) with the 97% similarity.

### *assA* and *mcrA* genes analysis

Alkylsuccinate synthase gene (*assA*) and methyl coenzyme-M reductase gene (*mcrA*) as the key functional genes involved in the methanogenic *n*-alkane degradation process were investigated. PCR primer sets of assA2F/assA2R (Callaghan et al. 2010) and MLF/MLR (Luton et al. 2002) were used for the PCR amplification of *assA* and *mcrA* gene, respectively. PCR cycling conditions for both *assA* and *mcrA* gene were conducted as follows: 95 °C for 3 min; 40 cycles of 95 °C for 45 s, 55 °C for 60 s, 72 °C for 2 min; and 72 °C for 10 min. PCR products were purified and cloned, and positive clones were picked for sanger sequencing on ABI 377 automated sequencer (Liang et al. 2015). The valid nucleotide sequences were translated to protein sequences using ORFfinder translation tool (https://www.ncbi.nlm.nih.gov/orffinder/). Protein sequences were classified to OTUs using CD-HIT Suite (Huang et al. 2010) with the 97% similarity. Representative protein sequences were compared with GenBank Database using BLAST to identify similar sequences. Phylogenetic analyses were conducted using MEGA6.0 software with neighbor-joining method and 1000 bootstrap replicates.

### Data availability

The sequences generated in this study were deposited in GenBank under accession numbers SAMN08904491 and SAMN08904496 (bacterial and archaeal 16S rRNA genes), MH192396-MH192461 (*assA* genes), MH192647-MH192713 (*mcrA* genes). The sequencing data of alkane-free control cultures were available as previously (Ji et al. 2019).

## Results

### Methane and intermediate metabolites analysis

**Fig. 1:**
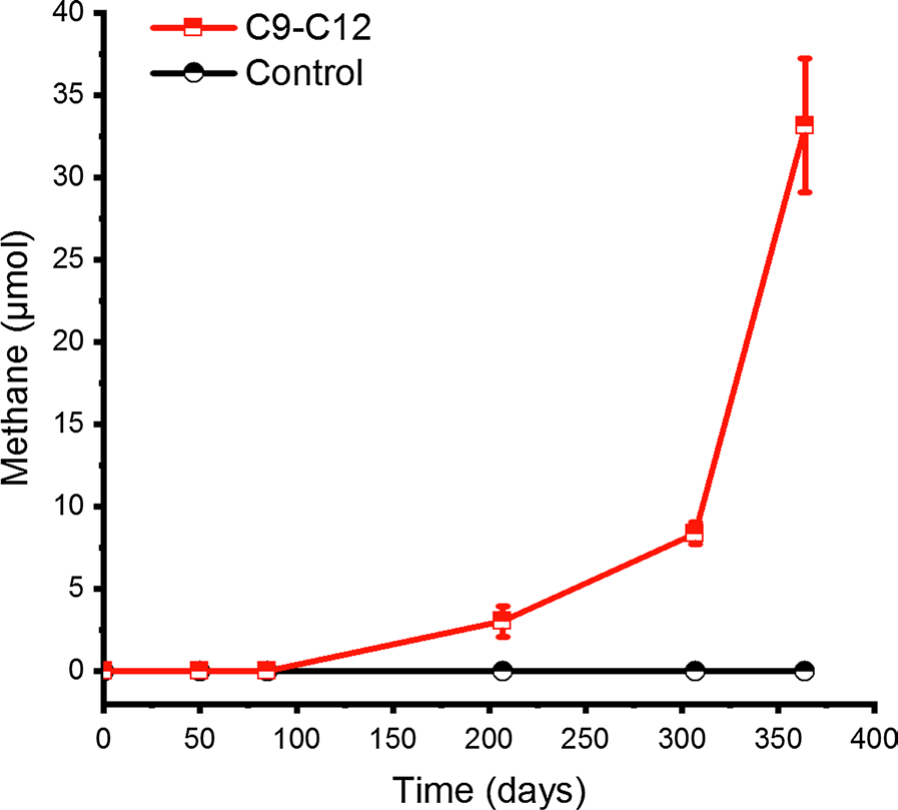
Methane produced over time in the methanogenic enrichment cultures amended with a mixture of *n*-alkanes (C_9_–C_12_) and control cultures without alkane (Control). Date points are averages of measurements from duplicate cultures and bars indicate standard deviations

Methane production started after 85 days’ incubation in alkane-amended (a mixture of C_9_ to C_12_*n*-alkanes) enrichment cultures and reached about 33 μmol at the end of the incubation (364 days) (Fig. 1). No methane was detected in the alkane-free controls (Ji et al. 2019) (Fig. 1).

**Fig. 2:**
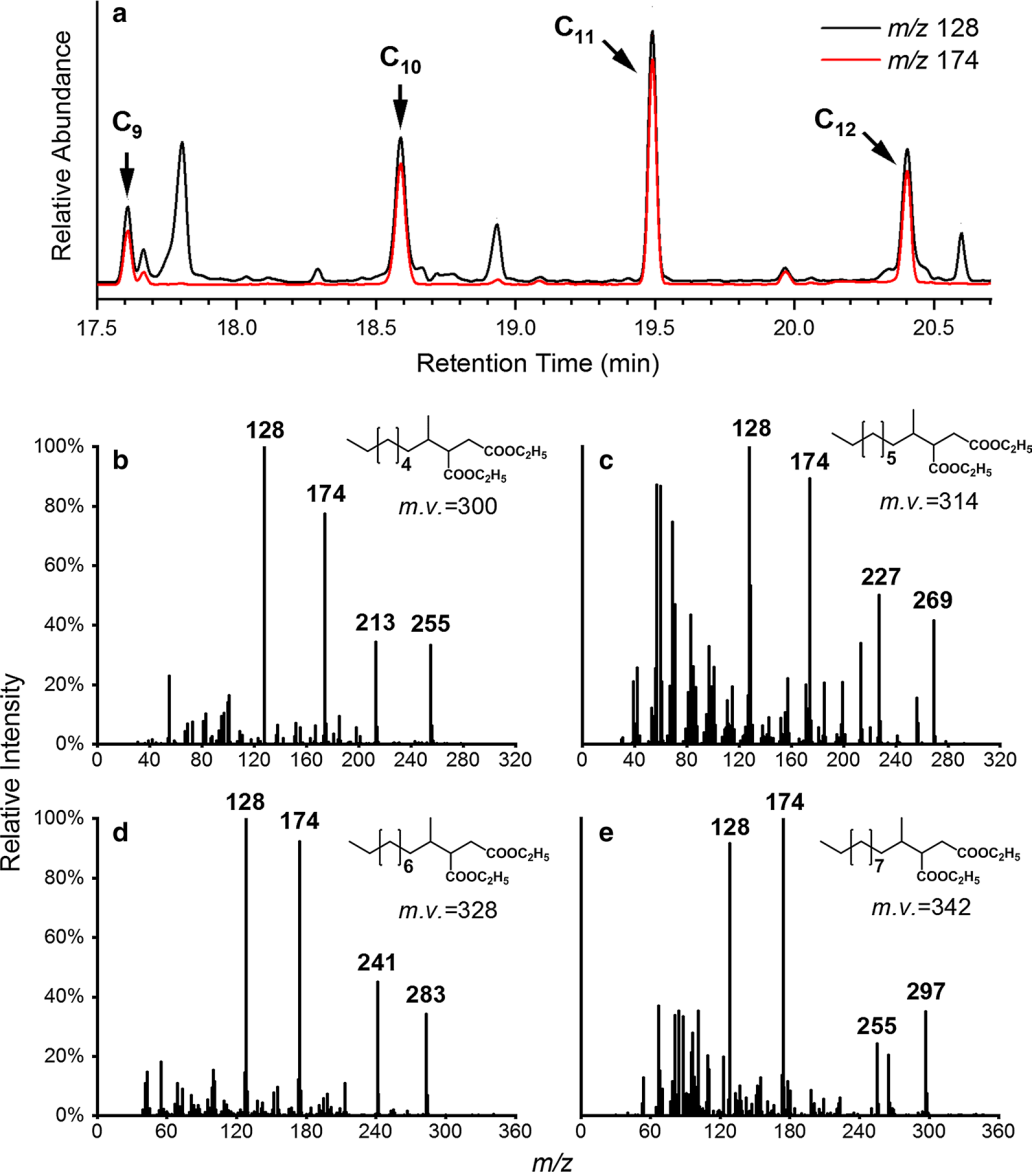
Detection of putative alkylsuccinates in the alkane-amended enrichment cultures. **a** Partial GC–MS *m/z* 128 and *m/z* 174 selected ion chromatogram showing the presence of (1-methyloctyl)succinate (C_9_), (1-methylnonyl)succinate (C_10_), (1-methyldecyl)succinate (C_11_) and (1-methylundecyl)succinate (C_12_). **b** Mass spectral profiles of (1-methyloctyl)succinate. **c** Mass spectral profiles of (1-methylnonyl)succinate. **d** Mass spectral profiles of (1-methyldecyl)succinate. **e** Mass spectral profiles of (1-methylundecyl)succinate

Potential anaerobic intermediates of *n*-alkanes were analyzed by GC–MS. (1-Methyloctyl)succinate (C_9_ alkylsuccinate), (1-methylnonyl)succinate (C_10_ alkylsuccinate), (1-methyldecyl)succinate (C_11_ alkylsuccinate) and (1-methylundecyl)succinate (C_12_ alkylsuccinate), generated from fumarate addition to the alkanes *n*-nonane, *n*-decane, *n*-undecane and *n*-dodecane respectively, were detected in the alkane-amended enrichment cultures (Fig. 2). All identified metabolites displayed the signature fragments at *m/z* 128, 174, [M–45]^+^ and [M–87]^+^, which are distinctive for alkylsuccinates (Bian et al. 2014) (Fig. 2). The identity of C_9_ alkylsuccinate in the alkane-amended enrichment cultures was confirmed by comparing its ion fragmentation patterns and retention time with that of a synthesized standard (Fig. 2, Additional file 1: Figure S1). No alkylsuccinates were identified in the alkane-free controls (Ji et al. 2019).

Long-chain fatty acids included tetradecanoate, pentadecanoate, hexadecanoate, heptadecanoate, octadecanoate, eicosanoate and docosanoate were detected in the alkane-amended enrichment cultures (Additional file 1: Figure S3). Only hexadecanoate and octadecanoate were detected in the alkane-free controls. Several α,ω-dicarboxylic acids were specifically identified in the alkane-amended enrichment cultures by comparing to the authentic standards with mass spectral profiles and retention times. These included butanedioic (succinic) acid, pentanedioic (glutaric) acid, hexanedioic (adipic) acid, octanedioic (suberic) acid and nonanedioic (azelaic) acid (Additional file 1: Figure S2).

### Microbial community compositions

**Fig. 3:**
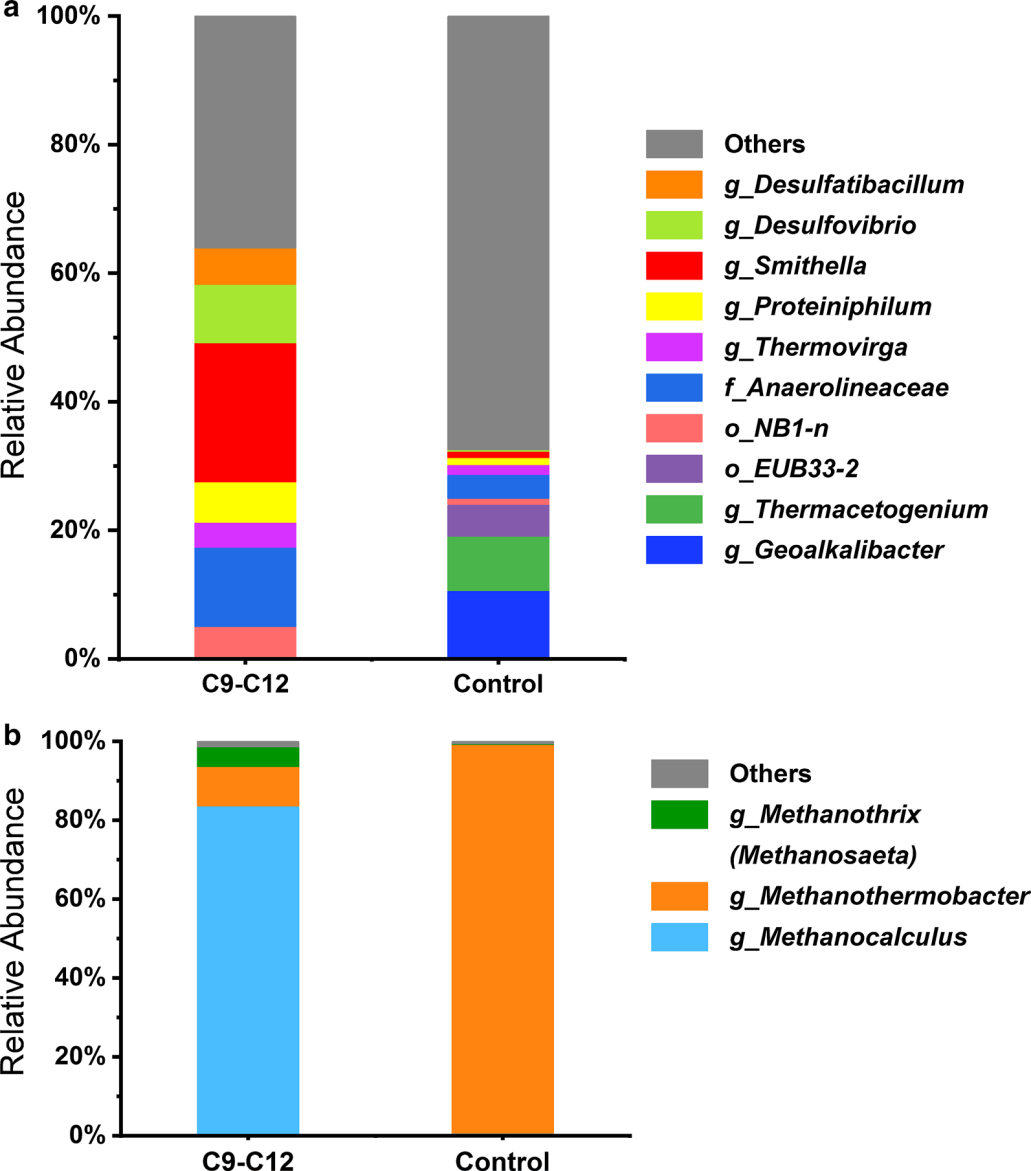
Microbial community compositions in the alkane-amended enrichment cultures and alkane-free control cultures as determined by Illumina sequencing of 16S rRNA genes. **a** Bacterial population in both cultures. Sequences comprising more than 3% in at least one culture were shown. **b** Archaeal population in both cultures. Sequences comprising more than 1% in at least one culture were shown. The notations in the legend g, f, and o stand for the OTUs assigned to genus, family and order levels, respectively

Substantial difference of microbial community compositions was observed between alkane-amended enrichment cultures and alkane-free control cultures at the end of the incubation (364 days). *Smithella* sp. had the highest relative abundance in the alkane-amended enrichment cultures (Fig. 3a). Other abundant bacterial phylotypes affiliated to *Anaerolineaceae*, *Desulfovibrio*, *Desulfatibacillum*, *Proteiniphilum*, *Thermovirga*, and unclassified *NB1*-*n* (Fig. 3a). In the alkane-free control cultures, members of *Geoalkalibacter* and *Thermacetogenium* became the dominant bacteria (Ji et al. 2019) (Fig. 3a). The archaeal community in the alkane-amended enrichment cultures was dominated by hydrogenotrophic methanogens of *Methanocalculus* (84%) and *Methanothermobacter* (10%) (Fig. 3b). *Methanothrix* (*Methanosaeta*, acetoclastic methanogen) was also detected in the alkane-amended enrichment cultures and comprised about 5% of the total archaeal community (Fig. 3b). The archaeal community in the alkane-free control cultures was essentially comprised by *Methanothermobacter* (98%) (Ji et al. 2019) (Fig. 3b).

### Phylogenetic analysis of *assA* and *mcrA* genes

**Fig. 4:**
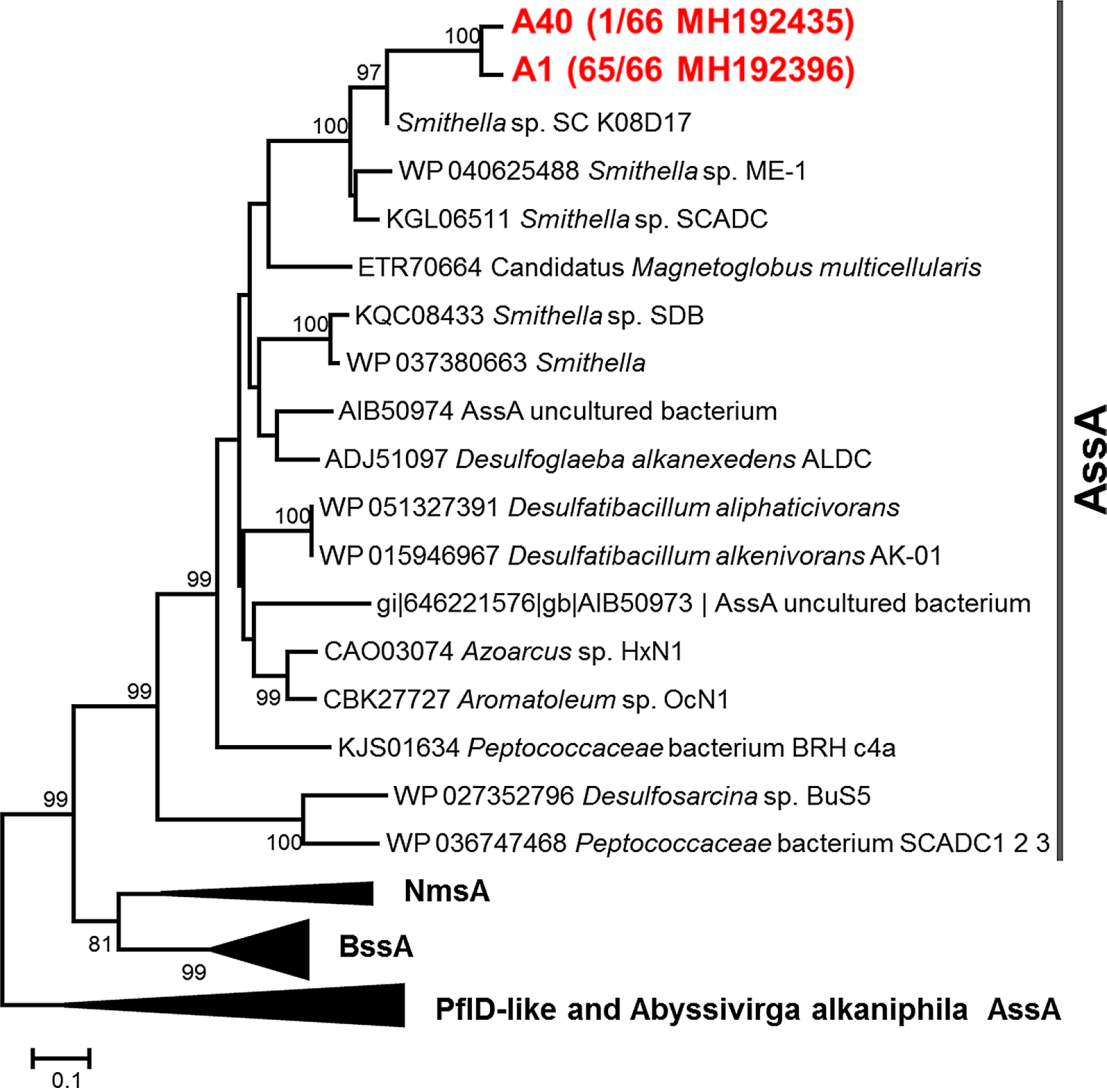
Phylogenetic tree of alkylsuccinate synthase alpha-subunit (*assA*) amino acid sequences obtained from alkane-amended enrichment cultures. The topology of the tree was obtained using the neighbor-joining method and performing 1000 bootstrap replicates (values below 75% are not shown)

Genes encoding for the alkylsuccinate synthase were only detected in the alkane-amended enrichment cultures. All sequences were clustered into *Smithella* subclade and were most closely related with *assA* sequence of *Smithella* sp. SC_K08D17 (Fig. 4). Both alkane-amended enrichment cultures and control cultures detected *mcrA* genes (Ji et al. 2019). In the alkane-amended enrichment cultures, most *mcrA* sequences affiliated with *Methanocalculus* and only one sequence (a total of 67 valid sequences) belonged to *Methanothermobacter* (Additional file 1: Figure S4).

## Discussion

### Methanogenic biodegradation of C_9_ to C_12_*n*-alkanes initiated by addition to fumarate

The detection of corresponding fumarate addition products (C_9_ to C_12_ alkylsuccinates) provides convincing evidence that the oxidation of C_9_ to C_12_*n*-alkanes was initiated by addition to fumarate under methanogenic conditions. It is supported further by the detection of *assA* genes. Previous studies have reported C_1_ to C_8_ alkylsuccinates detected in the production water from oil reservoirs (Agrawal and Gieg 2013; Bian et al. 2015; Duncan et al. 2009; Gieg et al. 2010). And C_1_ to C_9_, C_15_ to C_20_ alkylsuccinates have been detected under methanogenic conditions associated with microorganisms derived from oil reservoirs (Qin et al. 2017; Toth and Gieg 2017). Here the identification of C_9_ to C_12_ alkylsuccinates fills a gap that a series of *n*-alkanes can be activated by fumarate addition by oilfield-related microorganisms.

Except for fumarate addition products, several dicarboxylic acids were detected in alkane-amended enrichment cultures. These diacids may be cell-associated or secreted from the cells. Oberding and Gieg detected several α,ω-dicarboxylic acids in methanogenic *n*-octacosane-degrading enrichment cultures (Oberding and Gieg 2018). The authors suggested that these dicarboxylic acids might act as biosurfactants, which could increase substrate accessibility (Oberding and Gieg 2018). Although the origin of these dicarboxylic acids is elusive, the fatty acids as the downstream metabolites involved in fumarate addition pathway (Wilkes et al. 2002), may play a role in alkane emulsification in the current study (Embree et al. 2014).

### Key members involved in methanogenic *n*-alkane degradation

The dominant bacteria in the alkane-amended enrichment cultures were *Smithella*. Members of *Smithella* have been detected in numerous methanogenic alkane- and crude oil-degrading enrichment cultures (Cheng et al. 2013; Oberding and Gieg 2018; Siddique et al. 2011; Wawrik et al. 2016; Zengler et al. 1999) and are generally considered as syntrophic *n*-alkane degraders (Gray et al. 2011). In this study, *assA* genes closely related to *Smithella* species was detected, suggesting that *Smithella* participated in methanogenic *n*-alkane degradation and initiated alkane activation by fumarate addition reaction.

The abundance of *Anaerolineaceae* was found to be increased in the alkane-amended enrichment cultures. Microorganisms affiliated to the family *Anaerolineaceae* have been detected in a vast number of methanogenic alkane-degrading enrichment cultures and were implicated to be responsible for alkane activation in these cultures (Liang et al. 2015; Liang et al. 2016; Mohamad Shahimin et al. 2016; Mohamad Shahimin and Siddique 2017). However, *Anaerolineaceae*-related *assA* genes were not detected in the current culture, consistent with previous studies (Liang et al. 2016; Mohamad Shahimin et al. 2016). It has also been suggested that *Anaerolineaceae* may serve as a secondary degrader in oxidizing fermentative products from primary degraders (Tan et al. 2015). Based on the results of this study, the role of *Anaerolineaceae* is currently unknown.

Our results suggest that fumarate addition is a key alkane initial activation mechanism under methanogenic conditions. *Smithella* were identified as primary syntrophic *n*-alkane degraders, which can activate C_9_ to C_12_*n*-alkanes by addition to fumarate. This work expands our knowledge about the biochemical process involved in the methanogenic hydrocarbon biodegradation in petroleum reservoirs and oil-contaminated environments.

## Supplementary information

**Additional file 1: Figure S1.** GC–MS analysis of a diethyl 2-(1-methyloctyl)succinate (C_9_ alkylsuccinate) standard. (a) GC partial ion chromatogram following selection for the *m/z* 128 ion of a diethyl 2-(1-methyloctyl)succinate standard, (b) Mass spectral profiles of diethyl 2-(1-methyloctyl)succinate (retention time, 17.60 min). **Figure S2.** Mass spectral profiles of dicarboxylic acids identified in alkane-amended enrichment cultures. Left panel: compound detected in the alkane-amended enrichment cultures. Right panel: ethyl-derivatized authentic standards. **Figure S3.** Mass spectral profiles of fatty acids (ethyl derivatives) identified in alkane-amended enrichment cultures. **Figure S4.** Phylogenetic tree of deduced amino acid sequences of methyl coenzyme-M reductase genes (*mcrA*) from alkane-amended enrichment culture (*in red*). Topology of the tree was obtained by the neighbor-joining method. Bootstrap values (n = 1000 replicates), values below 75% are not shown.

## Data Availability

Raw reads from microbial community sequencing are available in the GenBank archive at the National Center for Biotechnological Information (NCBI) as listed in the manuscript.

## Abbreviations

*assA*: alkylsuccinate synthase gene
*mcrA*: methyl coenzyme-M reductase gene
C_9_ alkylsuccinate: (1-methyloctyl)succinate
C_10_ alkylsuccinate: (1-methylnonyl)succinate
C_11_ alkylsuccinate: (1-methyldecyl)succinate
C_12_ alkylsuccinate: (1-methylundecyl)succinate
