# Supplementary material for: Methanogenic biodegradation of C9 to C12n-alkanes initiated by Smithella via fumarate addition mechanism
Source: AMB Express. 2020 Feb 1;10:23. doi: 10.1186/s13568-020-0956-5 (PMC6995468; doi:10.1186/s13568-020-0956-5)
Supplement: Supplementary file 1 — Additional file 1: Figure S1. GC–MS analysis of a diethyl 2-(1-methyloctyl)succinate (C9 alkylsuccinate) standard. (a) GC partial ion chromatogram following selection for the m/z 128 ion of a diethyl 2-(1-methyloctyl)succinate standard, (b) Mass spectral profiles of diethyl 2-(1-methyloctyl)succinate (retention time, 17.60 min). Figure S2. Mass spectral profiles of dicarboxylic acids identified in alkane-amended enrichment cultures. Left panel: compound detected in the alkane-amended enrichment cultures. Right panel: ethyl-derivatized authentic standards. Figure S3. Mass spectral profiles of fatty acids (ethyl derivatives) identified in alkane-amended enrichment cultures. Figure S4. Phylogenetic tree of deduced amino acid sequences of methyl coenzyme-M reductase genes (mcrA) from alkane-amended enrichment culture (in red). Topology of the tree was obtained by the neighbor-joining method. Bootstrap values (n = 1000 replicates), values below 75% are not shown. [file 13568_2020_956_MOESM1_ESM.docx]

**Additional file**

**Methanogenic biodegradation of C_9_ to C_12_ *n*-alkanes initiated by *Smithella* via fumarate addition mechanism**

Jia-Heng Ji^1, †^, Lei Zhou^1, †^, Serge Maurice Mbadinga^1^, Muhammad Irfan^1, 2^, Yi-Fan Liu^1^, Pan Pan^1^, Zhen-Zhen Qi^1^, Jing Chen^1^, Jin-Feng Liu^1^, Shi-Zhong Yang^1^, Ji-Dong Gu^3^, Bo-Zhong Mu^1, 4, *^

^1^*State Key Laboratory of Bioreactor Engineering and School of Chemistry and Molecular Engineering, East China University of Science and Technology, 130 Meilong Road, Shanghai 200237, P.R. China*

^2^*Department of Chemical, Polymer and Composite Materials Engineering, University of Engineering and Technology, KSK Campus, Lahore 54890, Pakistan*

^3^*School of Biological Sciences, The University of Hong Kong, Pokfulam Road, Hong Kong Special Administrative Region, P.R. China*

^4^*Engineering Research Center of Microbial Enhanced Oil Recovery, East China University of Science and Technology, 130 Meilong Road, Shanghai 200237, P.R. China*

^*^Corresponding Author:

Bo-Zhong Mu: E-mail: bzmu@ecust.edu.cn; Phone: +86 21 64252063; Fax: +86 21 64252485

^†^These authors contributed equally to this work.





**Figure S1.** GC-MS analysis of a diethyl 2-(1-methyloctyl)succinate (C_9_ alkylsuccinate) standard. (a) GC partial ion chromatogram following selection for the *m/z* 128 ion of a diethyl 2-(1-methyloctyl)succinate standard, (b) Mass spectral profiles of diethyl 2-(1-methyloctyl)succinate (retention time, 17.60 min).

**
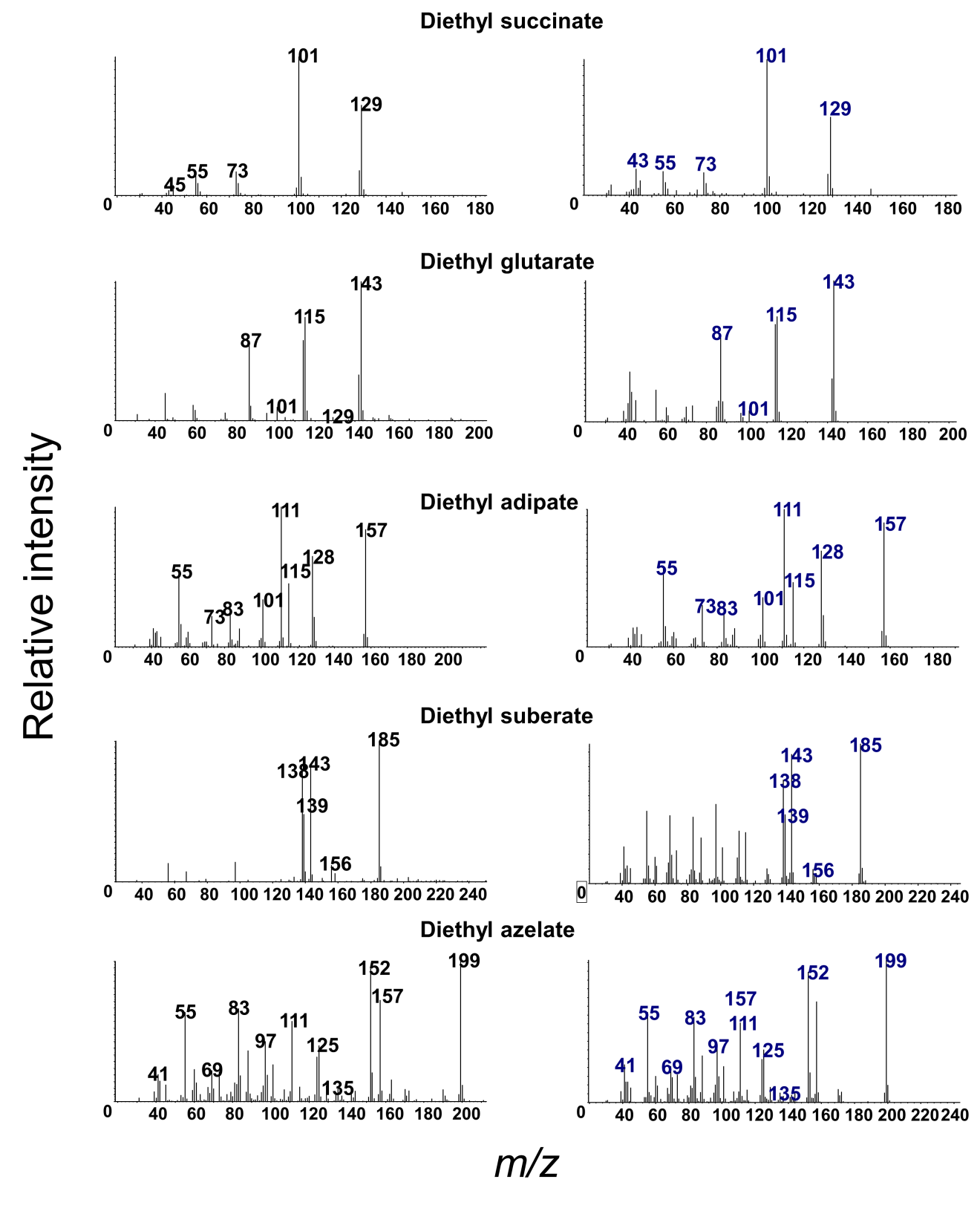
**

**Figure S2.** Mass spectral profiles of dicarboxylic acids identified in alkane-amended enrichment cultures. Left panel: compound detected in the alkane-amended enrichment cultures. Right panel: ethyl-derivatized authentic standards.

**
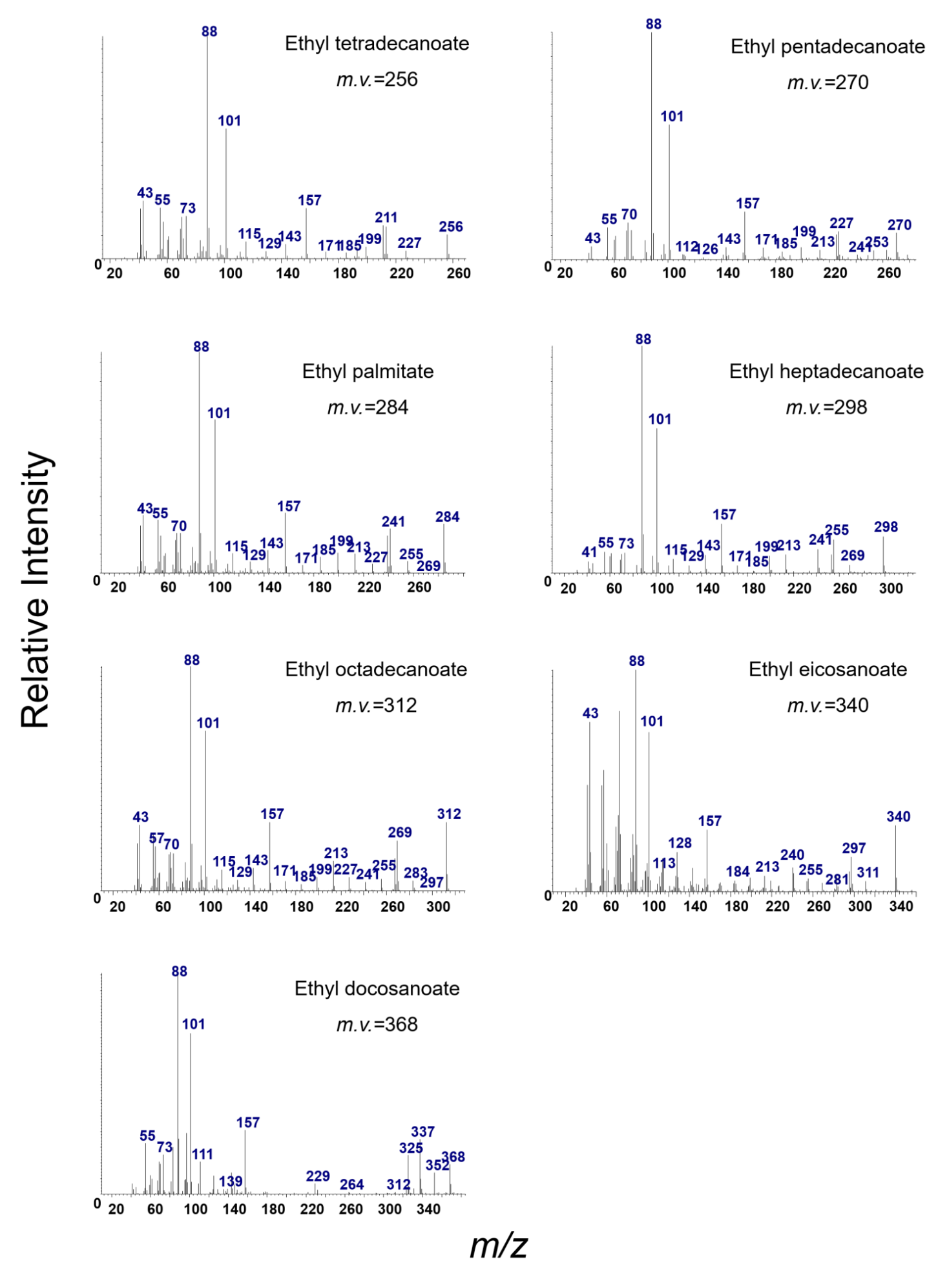
**

**Figure S3.** Mass spectral profiles of fatty acids (ethyl derivatives) identified in alkane-amended enrichment cultures.

**
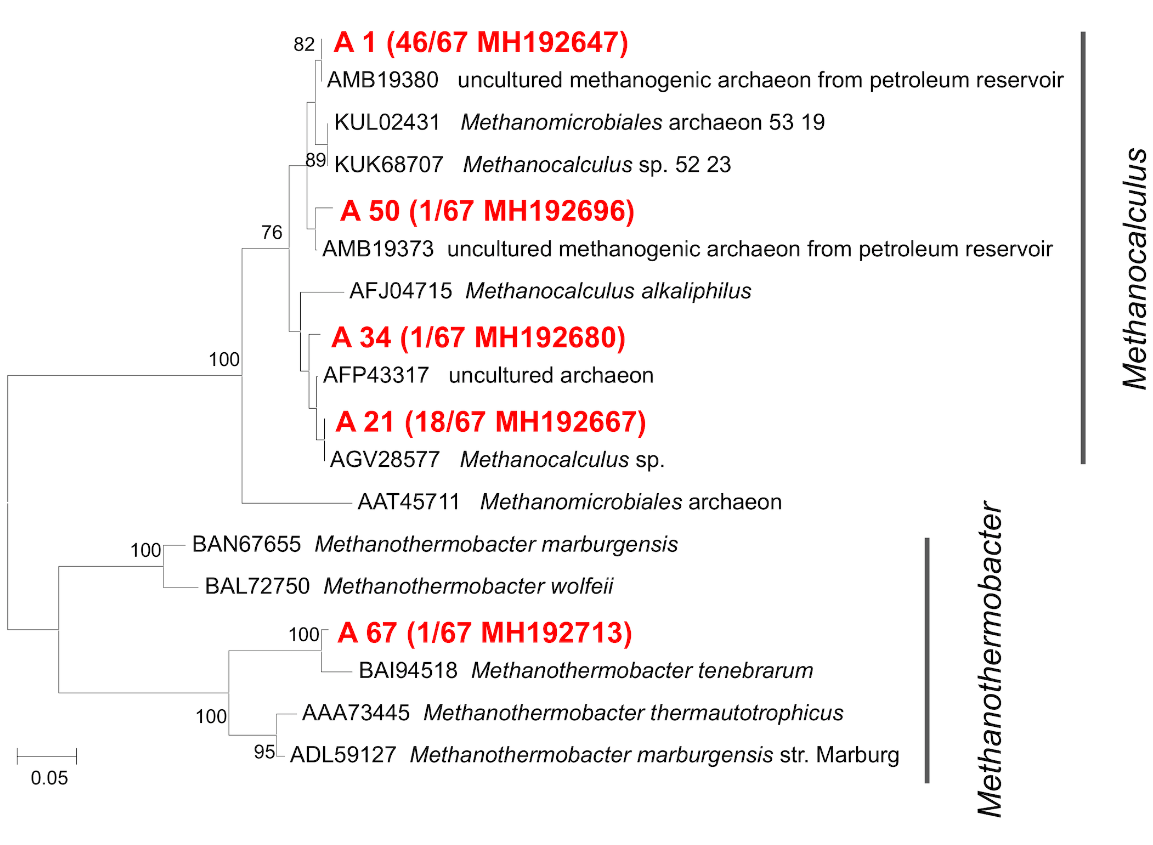
**

**Figure S4.** Phylogenetic tree of deduced amino acid sequences of methyl coenzyme-M reductase genes (*mcrA*) from alkane-amended enrichment culture (*in red*). Topology of the tree was obtained by the neighbor-joining method. Bootstrap values (n=1,000 replicates), values below 75% are not shown.
